# Margins of beneficial daily dosage of supplements in prevention of COVID-19

**DOI:** 10.17179/excli2021-3790

**Published:** 2021-04-26

**Authors:** Vladimir Ajdžanovic, Branko Filipovic, Branka Šošic-Jurjevic, Marko Miler, Verica Miloševic

**Affiliations:** 1 Department of Cytology, Institute for Biological Research "Siniša Stankovic" - National Institute of Republic of Serbia, University of Belgrade, Belgrade, Serbia

## ⁯

***Dear Editor,***

Over the past year, mankind has experienced the rapid and unrestrainable spread of severe acute respiratory syndrome coronavirus 2 (SARS-CoV-2), which caused the coronavirus disease 2019 (COVID-19) pandemic and globally challenged healthcare systems. SARS-CoV-2 is a large RNA virus that targets cells expressing the angiotensin-converting enzyme 2 receptor, specifically lung airway- or alveolar- epithelial cells, vascular endothelial cells and macrophages, as well as myocardial and renal cells (Tay et al., 2020[42]; Bermano et al., 2021[7]). Massive inflammatory response (cytokine storm - the release of pro-inflammatory cytokines and chemokines) is a complication that follows severe cases of SARS-CoV-2 infection, which are most frequent in the older population, and results in serious damage of the lung epithelium (Tay et al., 2020[42]; Becerra-Muñoz et al., 2021[6]). Broad symptomatology of the infection includes impaired smell and taste, runny nose, fever, sore throat, cough, dyspnea, fatigue and hypercoagulability, with the tendency of developing pneumonia or myocarditis (Wessels et al., 2020[47]; Krumm et al., 2021[29]). In line with this, the cytokine storm may spread through the human body and provoke multi-organ collapse and death (Ruan et al., 2020[38]). Obviously, globally available, cheap and safe preventive or therapeutic options, with minimal side effects and simple application, are desired during the present pandemic (Wessels et al., 2020[47]). Supplementation with micronutrients, such as vitamins and minerals, has gained an increasing interest, either in the context of prevention or as a part of the remediation of COVID-19 (Abobaker et al., 2020[1]; Alexander et al., 2020[2]). Alternatives for fighting COVID-19 become even more important keeping in mind the lack of consensus pertinent to the application of antiviral drugs or the global vaccination doctrine that is still developing (Hoang et al., 2020[18]).

**Figure 1:**
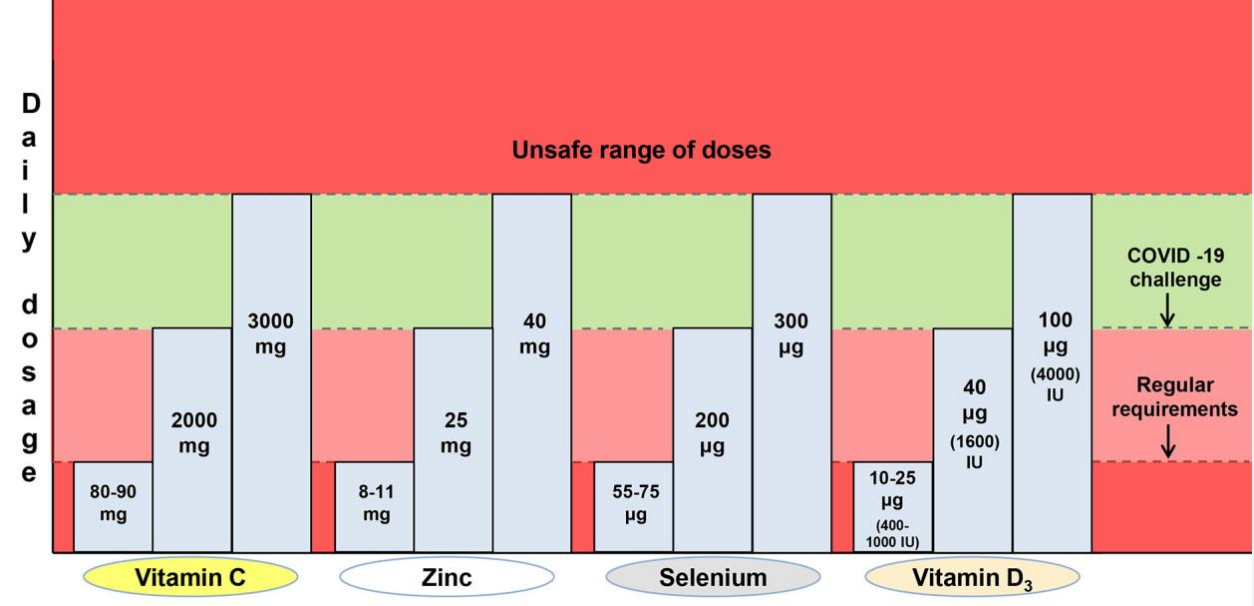
The margins of beneficial daily dosage of four commonly supplemented micronutrients (vitamin C, zinc, selenium and vitamin D3) in the prevention of COVID-19

Deficiencies of vitamin C, zinc, selenium or vitamin D, which are frequent during advanced age, contribute to age-related diseases including immunodeficiencies, metabolic and cardiovascular diseases (Jayachandran et al., 2000[23]; Holmberg et al., 2017[22]; Bjørklund et al., 2020[8]; Alexander et al., 2020[2]). Some age-related diseases as well as ageing *per se* are characterized by signs of low-grade inflammation, while all these represent aggravating factors in the context of COVID-19 (Sanada et al., 2018[39]; Alexander et al., 2020[2]). Sufficient levels of these micronutrients are essential for adequate immunocompetence and resistance to COVID-19 development, especially in the older population. In this brief normative work, from the domain of public health, we shall define the margins of beneficial daily dosage of four commonly supplemented micronutrients (vitamin C, zinc, selenium and vitamin D_3_) in the prevention of COVID-19 (Table 1(Tab. 1); Figure 1(Fig. 1)). This implies determining both the lowest sufficient doses that may respond to COVID-19 challenge as well as the highest safe dosage of the supplementation. The beneficial dose range of oral nutritional supplements is still insufficiently elaborated in general, while commercial products are often sub-optimally dosed. So, we will not go into details regarding the mechanisms of action of these micronutrients in COVID-19 prevention, though the directions for good praxis of their application will be highlighted.

Vitamin C (ascorbic acid) is an essential water-soluble micronutrient. It acts as a cofactor for numerous enzymes and facilitates the production of catecholamines, glucocorticoids, vasopressin, L-carnitine and collagen, which are crucial for cellular function and homeostasis (Carr and Maggini, 2017[11]; Hoang et al., 2020[18]). Given that it is not synthesized by primates, humans are dependent on the nutritional intake of vitamin C. The requirement of 90 mg/day for men and 80 mg/day for women ensures a normal plasma level of 50 μM/L, but this is insufficient when a subject is under stress or viral exposure (Holford et al., 2020[20]). It was observed that the majority of critically ill COVID-19 patients express hypovitaminosis C, with mean plasma levels of 29 μM/L in survivors and 15 μM/L in non-survivors (Arvinte et al., 2020[3]). Vitamin C was demonstrated to manifest antiviral, anti-inflammatory, immunomodulating, antioxidant and antithrombotic properties (Marik, 2018[32]; Colunga Biancatelli et al., 2020[12]; Holford et al., 2020[20]). Cytokine downregulation upon vitamin C application may contribute to 'calming' the COVID-19 - characteristic cytokine storm (Holford et al., 2020[20]), while high doses of vitamin C could reduce the need for other drugs (corticosteroids or antivirals), which are toxic under certain conditions (Hoang et al., 2020[18]). United States nutritional recommendations state that the tolerable upper limit of vitamin C for adults is 2 g/day (Abobaker et al., 2020[1]). On the other hand, it is suggested that the daily dose of vitamin C as a preventive agent should be started at > 2 g (Hoang et al., 2020[18]). According to the European Food Safety Authorities and the gastrointestinal criteria taken into account, the lowest observable adverse effect dose of vitamin C is 3-4 g/day (Holford et al., 2020[20]). High doses of vitamin C (10 g/day) have been shown to increase the risk of oxalate nephropathy and oxalate kidney stones (Nabzdyk and Bittner, 2018[35]). Individuals with glucose-6-phosphate dehydrogenase deficiency should avoid high doses of vitamin C due to potential acute hemolysis (Rees et al., 1993[37]). Also, patients suffering from cardiovascular issues, who are taking anticoagulant aspirin therapy, may have impeded gastrointestinal absorption of vitamin C (Basu, 1982[5]). Although the oral doses of vitamin C that reach even 8 g/day are proposed to reduce the incidence and duration of respiratory infections (Holford et al., 2020[20]), all the above-mentioned data suggest that the dose range between 2 and 3 g/day could represent the rational choice for COVID-19 prevention in healthy individuals (Table 1(Tab. 1); Figure 1(Fig. 1)).

After iron, zinc (Zn) is the second most common trace element present in the human organism. It impacts key aspects of the immune system, through mediation of basic cellular functions like DNA replication, RNA transcription, cell division, apoptosis, membrane stabilization and antioxidative defense (Joachimiak, 2021[24]). More specifically, zinc maintains the skin's barrier functionality, regulates gene expression in lymphocytes and is responsible for normal development and function of neutrophils and natural killer cells (nonspecific immunity players) (Shankar and Prasad, 1998[41]; Joachimiak, 2021[24]). Zinc deficiency results in dysfunctional humoral and cell-mediated immunity, while serum zinc values of < 0.7 mg/L in the elderly were found to increase the risk of pneumonia (Tuerk and Fazel, 2009[44]; Barnett et al., 2010[4]; Alexander et al., 2020[2]). Generally, long-term zinc deficiency increases production of inflammatory biomarkers (Bonaventura el al., 2015[9]). Probably the most important fact in the COVID-19 context is that zinc deficiency causes lymphopenia and increased apoptosis of lymphocytes, all leading to immunodeficiency (Kolenko et al., 2001[28]). Jothimani et al. (2020[25]) have recently reported that COVID-19 patients have significantly lower zinc levels in comparison with healthy individuals and, more importantly, this is associated with increased risk of developing complications. Given that zinc is known to decrease the production of inflammatory cytokines and oxidative stress biomarkers (Prasad, 2014[36]), it is rationale to believe that upon adequate application it may mitigate the cytokine storm and further deteriorating course of COVID-19. The recommended dietary intake of zinc is 11 mg/day for men and 8 mg/day for women, with a tolerable upper intake level of 40 mg/day for both sexes (Mossink, 2020[34]). The same author suggests that zinc dosages considered as safe for oral supplementation in adults are between 10 and 12 mg/day (Mossink, 2020[34]). When zinc is used as a preventive agent during prolonged periods, an intake of ≤ 25 mg/day is recommended, with a warning that high intake of zinc may disturb the copper balance (Alexander et al., 2020[2]). In estimating beneficial zinc doses related to COVID-19, some cases of recovered patients upon the application of even 115-184 mg Zn/day for 10-14 days (Finzi, 2020[15]) shouldn't be disregarded. Overall, a dose range between 25 and 40 mg Zn/day appears to be safe and promising in the prevention of COVID-19 (Table 1(Tab. 1); Figure 1(Fig. 1)). In parallel, periodic checking of the serum copper status is advised.

Selenium (Se) is an essential trace element for the redox processes in humans, existing in the form of selenocysteine in catalytical centers of many selenoproteins (Fairweather-Tait et al., 2011[14]). This unique element (the only one specified in the genetic code) is well recognized due to its capability of reducing the incidence and severity of various viral infections (Zhang et al., 2020[48]). It is essential at rather low levels of intake (55-75 μg/day) and modulates the molecular mechanisms of inter-linked redox homeostasis, stress response and inflammatory cascade (Bermano et al., 2020[7]; Zhang et al., 2020[48]). Nutrition intake of selenium usually depends on its level in the soil and sub-optimal blood selenium status (≤ 85 μg/L or ≤ 100 μg/L, depending on the literature source) is common in human populations worldwide (Alexander et al., 2020[2]; Bermano et al., 2020[7]). It has been recently observed that the selenium levels in serum are significantly higher in surviving COVID-19 patients in comparison with non-survivors (Moghaddam et al., 2020[33]). Interleukin 6, that is released in high quantities during a cytokine storm in critically ill COVID-19 patients and leads to lung inflammation, was reported to be downregulated by selenium (Hoffmann and Berry, 2008[19]; Conti et al., 2020[13]). So, in the subjects with sub-optimal status, supplementation at a dose of 100-200 μg Se/day is recommended in the prevention of SARS-CoV-2 infection (Alexander et al., 2020[2]). United States Medical Authorities suggest that the tolerable upper limit of selenium is 400 μg/day, while toxic doses are considered to be above 800 μg/day (Zhang et al., 2020[48]). On the other hand, the European Union Scientific Committee on Food (European Commission; Brussels, Belgium, 2000) provided the opinion that a total long-term intake of selenium should not exceed 300 μg/day, given that higher intakes may be associated with toxicity (Alexander et al., 2020[2]). Critical analysis of above-elaborated literature data reliably borders a dose range between 200 and 300 μg Se/day as optimal in the prevention of COVID-19 (Table 1(Tab. 1); Figure 1(Fig. 1)).

Vitamin D_3_ (cholecalciferol) is a fat-soluble vitamin that is primarily produced in the skin, upon exposure to ultraviolet (UV) rays, or may be obtained from specific food (egg yolk, fish liver oil, fatty fishes, etc.) and supplementation products (Lips, 2006[30]; Uchitomi et al., 2020[45]). Its biological activity is conditioned by hydroxylation in the liver into 25-hydroxyvitamin D_3_ (calcidiol - the major circulatory form of vitamin D_3_), and subsequently in the kidney, into the physiologically active metabolite 1,25-dihydroxyvitamin D_3_ (calcitriol) (Lips, 2006[30]; Hill et al., 2018[17]; Uchitomi et al., 2020[45]; Alexander et al., 2020[2]). Besides its important role in calcium homeostasis and bone metabolism, vitamin D is known as a steroid hormone which stimulates maturation of immune cells (Alexander et al., 2020[2]; Hadizadeh, 2021[16]). Inverse proportions between blood levels of calcidiol and inflammatory biomarkers (c-reactive protein and interleukin 6) were observed (Liu et al., 2011[31]), while vitamin D treatment was found to reduce replication of respiratory viruses in bronchial epithelial cells *in vitro* (Telcian et al., 2017[43]). Plasma levels of calcidiol lower than 20 ng/mL define vitamin D insufficiency, while vitamin D deficiency starts at levels < 12.5 ng/mL (Brenner et al., 2017[10]). These statuses are frequent in people with limited access to sunlight or in ageing individuals with reduced synthesizing capacity (Holick, 2017[21]). In older adults, supplementation with 400-1000 IU vitamin D/day, along with a balanced diet and regular sun exposure, is advised to avoid deficiency (Shakoor et al., 2021[40]). In a large cohort of SARS-CoV-2 positive patients from the United States, an inverse relationship between circulating levels of calcidiol and virus positivity was observed, with higher positivity with calcidiol 'critical' values (< 20 ng/mL) and lower positivity with values of ≥ 50 ng/mL (Kaufman et al., 2020[27]). Generally, vitamin D deficient individuals have 4.6 times higher probability of developing COVID-19 than those with normal levels (Katz et al., 2021[26]). In a recently conducted trial, Wang and colleagues (2021[46]) observed that the application of 3200 IU vitamin D_3_/day decreased the rates of hospitalization and mortality in COVID-19 patients and, importantly, was beneficial in preventing infection among close household contacts. It is considered that in vitamin D deficiency, supplementation of 40 μg (1600 IU) vitamin D_3_/day could be beneficial for prevention of serious inflammation induced by SARS-CoV-2 (Alexander et al., 2020[2]) (Table 1(Tab. 1); Figure 1(Fig. 1)). Also, prolonged supplementation for preventive purposes should not exceed 100 μg (4000 IU) vitamin D_3_/day (Table 1(Tab. 1); Figure 1(Fig. 1)), with a view to avoid hypercalcemia, hypercalcuria and the eventual formation of renal stones (Alexander et al., 2020[2]).

Until the vaccination doctrine stabilizes globally, face mask utilization, physical distance, hygienic measures and optimal supplementation will represent mainstay measures in the prevention from COVID-19. Herein elaborated, optimal supplementation in the first place considers daily dosing with the supplements vitamin C, zinc, selenium and vitamin D_3_ within the benefit-to-risk window *i.e.* above the lowest sufficient dose that may respond to COVID-19 challenge and below the unsafe/toxic range of doses (Table 1(Tab. 1); Figure 1(Fig. 1)). The supplementation strategy should correspond to the subject's age, sex, ethnicity, health status, physical performance, hygienic-dietary habits, profession, level of stress exposure or frequency of social contacts, that all define COVID-19 susceptibility. Interindividual variability in responsiveness to the supplementation in terms of genetic or epigenetic factors may also exist. Given that the commercial products are often sub-optimally dosed, adjustments in this respect as well as efforts invested in designing multiple micronutrient-containing combinations would be welcome. Finally, for the desired results, supplement application in relation to a meal schedule/time of day should be in accordance with manufacturers' recommendation, while some periodic breaks in the supplementation are advised.

## Acknowledgements

This work was supported by the Ministry of Science, Education and Technological Development of the Republic of Serbia, via direct financing of our Institute (contract number: 451-03-9/2021-14/200007). We are grateful to Prof. Dr. Steve Quarrie, an English language professional, for his help in proofreading the manuscript.

## Conflict of interest

The authors declare that they have no conflict of interest.

## Figures and Tables

**Table 1:**
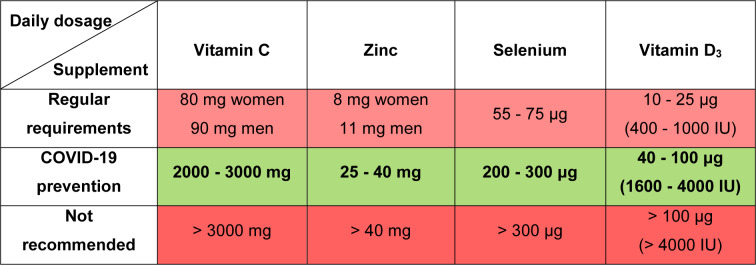
Daily dosage of vitamin C, zinc, selenium and vitamin D_3_ recommended under regular conditions and for COVID-19 prevention, as well as not recommended dose levels
